# Recent Advancements in Hydrogel Biomedical Research in Italy

**DOI:** 10.3390/gels10040248

**Published:** 2024-04-04

**Authors:** Eleonora Zanrè, Eva Dalla Valle, Edoardo D’Angelo, Francesca Sensi, Marco Agostini, Elisa Cimetta

**Affiliations:** 1 Department of Industrial Engineering (DII), University of Padova, 35131 Padova, Italy; eleonora.zanre@phd.unipd.it (E.Z.); eva.dallavalle@phd.unipd.it (E.D.V.); 2 Fondazione Istituto di Ricerca Pediatrica Città della Speranza (IRP), 35127 Padova, Italy; edoardo.dangelo@unipd.it (E.D.); francesca.sensi@studenti.unipd.it (F.S.); m.agostini@unipd.it (M.A.); 3 General Surgery 3, Department of Surgery, Oncology and Gastroenterology, University of Padova, 35122 Padova, Italy

**Keywords:** hydrogels, biomaterials, scaffolds, extracellular matrix, tissue engineering, drug delivery, regenerative medicine, 3D bioprinting, bioreactors, Italian research

## Abstract

Hydrogels have emerged as versatile biomaterials with remarkable applications in biomedicine and tissue engineering. Here, we present an overview of recent and ongoing research in Italy, focusing on extracellular matrix-derived, natural, and synthetic hydrogels specifically applied to biomedicine and tissue engineering. The analyzed studies highlight the versatile nature and wide range of applicability of hydrogel-based studies. Attention is also given to the integration of hydrogels within bioreactor systems, specialized devices used in biological studies to culture cells under controlled conditions, enhancing their potential for regenerative medicine, drug discovery, and drug delivery. Despite the abundance of literature on this subject, a comprehensive overview of Italian contributions to the field of hydrogels-based biomedical research is still missing and is thus our focus for this review. Consolidating a diverse range of studies, the Italian scientific community presents a complete landscape for hydrogel use, shaping the future directions of biomaterials research. This review aspires to serve as a guide and map for Italian researchers interested in the development and use of hydrogels in biomedicine.

## 1. Introduction

Hydrogels play a significant role in scientific research across various disciplines due to their unique properties and versatility. Different types of hydrogels, such as natural, synthetic, and hybrid, have been used to develop tissue-engineered constructs for applications in wound healing, organ transplantation, and drug delivery [1]. To give a comprehensive overview of their use and function, we first list and introduce the main applications of hydrogel-based materials.

The key to hydrogels’ successful applications in the field of biomaterials and tissue engineering is their ability to mimic the extracellular matrix (ECM), the natural environment of cells. By providing a three-dimensional (3D) structure that supports cell growth, proliferation, and differentiation, they play a fundamental role as scaffolds for tissue regeneration. Their high water content and porous structure resemble the native tissue environment, facilitating and regulating nutrients and gas diffusion to cells [2].

Hydrogels also see significant applications in wound healing and dressing due to their unique properties that promote wound closure and tissue regeneration. They create a moist environment that supports the natural healing process, prevents dehydration, and promotes cell migration, proliferation, and re-epithelialization [3,4].

Hydrogels have emerged as promising carriers for drug delivery due to their ability to encapsulate and release drugs in a controlled manner. These mechanisms are based on the regulation of their swelling and de-swelling in response to external stimuli such as temperature, pH, and light, all enabling targeted and sustained drug delivery, improving therapeutic outcomes, reducing side effects, and increasing patient compliance [5]. Similarly, they can encapsulate cells to protect them from immune response during transplantation, provide mechanical support, and create a conducive microenvironment for their survival and function. In general, hydrogel encapsulation greatly improves the delivery of cells into damaged tissues or organs, promoting tissue repair and regeneration [6].

More recent technological approaches, such as 3D bioprinting for the fabrication of complex tissue constructs, see hydrogels as the most widely used bioinks: printable and cell-friendly matrices enabling precise layer-by-layer deposition of cells and biomaterials to create tissue-like structures. The ability to incorporate cells, growth factors, and other bioactive components into the hydrogel bioink allows for the fabrication of functional tissues with tailored architectures [7].

Hydrogel-based biosensors and biomedical devices take advantage of the high biocompatibility and ease of functionalization to incorporate specific molecular recognition elements such as antibodies or enzymes, allowing them to detect and measure target analytes in complex biological samples. Their applications span from healthcare to environmental monitoring and food safety [8].

Hydrogels are also employed in the study of pathophysiological processes based on their capacity to closely mimic the ECM of living tissues. They can be used to create 3D environments that facilitate the study of cell interactions, behavior, and migration. This is crucial for understanding how cells respond to specific biochemical and mechanical cues within tissues. Hydrogels are employed to create in vitro models of the tumor microenvironment, allowing the study of cancer progression, invasion, and metastasis. These models help in understanding the complex interactions between cancer cells and the surrounding tissue [9].

The aim of this review is to provide a comprehensive overview of hydrogel research focusing on the applications, advancements, and contributions made by Italian researchers. We summarize and analyze the key findings, methodologies, and challenges, with a specific emphasis on tissue engineering, drug delivery, wound healing, regenerative medicine, and pathophysiological studies. By examining the diverse research areas and applications, as well as the scientific contributions and future directions, we seek to stimulate further research and collaborations in hydrogel-related fields within the Italian scientific community and abroad. We believe that fostering such connections could not only increase the exchange of knowledge and resources but also contribute to establishing the key role of Italian research in the field of hydrogels-based biomedicine within the broader international scientific community.

## 2. Hydrogel Characteristics and Classification

Hydrogels are gel-like materials with a strong affinity for water and are composed of interconnected polymer chains. They possess the unique ability to retain significant amounts of water within their structure without dissolving. This water-absorbing capacity derives from the hydrophilic functional groups attached to the polymer backbone, while the cross-links between the polymer chains contribute to the hydrogel’s resistance to dissolution [10,11]. The amount of water that each hydrogel can absorb and retain depends on factors such as the specific polymer used, as well as the type and density of the network connections. Generally, when in a swollen state, hydrogels contain a much higher mass fraction of water compared to the mass fraction of polymer [12].

Extracellular matrix (ECM) derived hydrogels will be here discussed first and separately from all other materials. This choice is due to the fact that their origin differs from other natural and synthetic hydrogels and their peculiar potential to more efficiently mimic the natural environment of in vivo tissues.

### 2.1. Hydrogels Derived from the ECM

The ECM is a constantly changing milieu that offers physical support to cells within tissues while also providing guidance signals for their proper functioning. The ECM primarily consists of glycosaminoglycans (GAGs) that are often covalently linked to proteins, forming proteoglycans, as well as other fibrous proteins. In vivo, these components are locally secreted by cells and spatially organized in a complex network constituting the ECM microenvironment. Each organ is characterized by a tissue-specific ECM composition, tailored to fulfill specific functions and emerging from the dynamic biological, physical, and chemical interactions between cellular components and their surrounding microenvironment during tissue development and disease progression [13].

Biological scaffolds derived from decellularized tissues have demonstrated efficacy in both pre-clinical animal studies and human clinical applications [14,15,16]. The process of cell removal from tissue structures should, at best, preserve the structural and functional proteins constituting the ECM. The obtained scaffolds vary significantly depending on the tissue sources, species of origin, decellularization techniques, and sterilization methods. These factors determine the composition and ultrastructure of the ECM, consequently impacting the biological response of engrafting cells in terms of cell migration, proliferation, and differentiation [17,18].

Decellularization is the process aimed at effectively removing all cellular and nuclear materials from a native tissue while preserving the composition, biological activity, and mechanical integrity of the ECM. Although of straightforward understanding, it should be highlighted how decellularization inevitably alters the native 3D architecture of the ECM. Commonly employed methods involve a combination of physical and chemical treatments. Physical treatments include agitation, sonication, mechanical massage, pressure, freezing, and thawing with the aim of disrupting cell membranes, facilitating the release of cell contents, and aiding in subsequent rinsing. Despite their utility, they typically result in partial decellularization and are thus often complemented with chemical treatments. Enzymatic digestion with trypsin or exposure to ionic solutions and detergents such as sodium dodecyl sulfate (SDS) or sodium deoxycolate (SDC) further chemically disrupts cell membranes and intercellular/extracellular connections.

The cellular material in the ECM is arranged in varying degrees of compactness depending on the tissue source. Consequently, during decellularization, adequate disruption of the ECM is crucial to ensure exposure of all cells to chaotropic agents and facilitate cellular material removal. Given the importance of mechanical compliance in determining the biological properties of the ECM, most decellularization processes aim to minimize such disruption [19]. The obtained materials must then undergo thorough testing to assess the absence of cellular materials and the preservation of the biological, biochemical, and biostructural properties of the ECM with respect to native tissue. Typical analyses to evaluate the decellularization efficacy include DNA extraction and quantification and cell nuclei counting using histology stains or immunofluorescence. Histology, immunohistochemistry, immunofluorescence, and scanning electron microscopy can be employed to investigate the distribution of proteins and the ultrastructural and topological organization [20].

Hydrogels derived from the decellularized extracellular matrix (dECM) are currently considered the most suitable option for replicating in vitro the in vivo-like tissue structure and composition. ECM-derived formulations provide a natural scaffolding microenvironment that enables reseeded cells to establish connections with the ECM and organize in 3D arrangements similar to those found in living tissues [21].

### 2.2. Non-ECM Hydrogels

Hydrogels can be classified based on the nature of their polymeric network, the origin of the material, fundamental chemistry, preparation details, and types of chemical bonding between building blocks. The three main categories are synthetic polymers, natural polymers, and hybrid materials, combining both synthetic and natural elements [12]. Commonly used synthetic polymers include poly(ethylene oxide) (PEO) and poly(ethylene glycol) (PEG) with a molecular mass below 20,000 g/mol, poly(2-hydroxyethyl methacrylate) (polyHEMA), poly(vinyl alcohol) (PVA), and poly(acrylamide) (PAAm) [11]. Natural polymers comprise proteins such as collagen and silk, denatured proteins like gelatin, and polysaccharides such as agar and alginate [22]. Hybrid materials form multi-component structures as grafted block copolymers or interpenetrating networks consisting of two or more independent polymers [23]. Additionally, degradable linkers can be incorporated into chemically stable hydrogels for controlled release or regenerative medicine applications [24]. Hybrid multi-component materials can be generated by grafting a biological motif, such as the RGD (arginine-glycine-aspartic acid) cell recognition sequence found in cell adhesive proteins like integrins, or a synthetic polymer to enhance cellular biocompatibility with the gel network [12].

The physical properties of gels mostly differ based on whether the polymer networks are chemically bonded or formed through physical, non-covalent interactions between polymer chains. Chemically cross-linked hydrogels typically exhibit enhanced structural integrity and mechanical properties compared to physically linked materials. The covalent cross-links formed through chemical reactions provide strong and durable connections between polymer chains, resulting in improved mechanical strength, stiffness, and stability. Chemically cross-linked hydrogels can thus withstand external forces and maintain structural integrity over extended periods, ensuring long-term functionality and performance and making them well-suited for applications such as tissue engineering, drug delivery systems, and biomedical devices [11,24,25].

On the other hand, physical gels have effective cross-link points that are connected by non-covalent bonds. In the simplest case, polymer chains loop around each other and form physical entanglement. The regions of polymer chain engagement at the effective cross-link points are typically larger than those in a single covalent point or a single chain entanglement. In some instances, physical gels exhibit more complex structures, such as helices, or in the case of algae-based gel alginate, divalent cations (such as Ca^2+^) form complexes that join the guluronic acid domains in the polysaccharide chains. The nature and definition of cross-link points fundamentally differ between chemical and physical gels, highlighting the distinct mechanisms by which they are formed [25].

### 2.3. Main Cross-Linking Methods

Cross-linking refers to the formation of chemical bonds between polymer chains within the hydrogel network. These bonds link the polymer chains, creating a 3D structure that gives the hydrogel its characteristic properties, such as elasticity and stability. Cross-linking can occur through various mechanisms, including chemical reactions or physical interactions. The growing interest in obtaining physically cross-linked hydrogels is primarily driven by the advantage of not requiring cross-linkers during synthesis.

Some methods to produce physically cross-linked hydrogels include physical entanglement, where polymer chains intertwine to form a 3D network. This method is common for gelatin-based and agarose hydrogels and can be achieved through techniques such as solution casting, solvent evaporation, or physical mixing of polymer chains [25]. Certain polymers undergo temperature-dependent phase changes from a soluble to a gel state, known as thermogelation. Pluronic^®^ copolymers are an example of this [10,21,26].

pH-induced gelation involves pH-sensitive polymers undergoing a conformational change in response to pH variations, leading to gelation. Alginate hydrogels, for instance, can be physically cross-linked by adjusting the pH of a polymer solution and adding divalent ions (i.e., calcium) under specific pH conditions [11,25].

Ionic gelation can be achieved by adding salts or other ions to polymer solutions, inducing the formation of ionic bonds between opposite charges in the polymer chains. Chitosan hydrogels, for example, can be physically cross-linked using multivalent cations such as tripolyphosphate ions [25,27,28].

Some polymers can self-assemble into organized structures like micelles or nanofibers, forming physically cross-linked hydrogels through molecular interactions such as hydrophobic or hydrogen bonding. Examples include self-assembling hydrogels based on peptides derived from collagen or elastin [25].

Photopolymerization is a process in which light or UV radiation initiates the polymerization and cross-linking of monomers or pre-polymers, offering precise control over the gelation process. This technique enables the fabrication of physically cross-linked hydrogels with spatial and temporal control. Photopolymerization involves exposing the monomers or pre-polymers to specific wavelengths of light, causing them to undergo chemical reactions and forming polymer networks. Furthermore, the versatility of photopolymerization allows for the incorporation of additives, such as drugs or growth factors, into the hydrogel matrix during the polymerization process, enhancing its functionality for specific applications [10].

The choice of method depends on the specific requirements of the hydrogel and the desired properties of the resulting material.

Similarly, there are several methods available for the preparation of chemically cross-linked hydrogels [25]. One common technique involves cross-linking through polymerization. Here, monomers undergo polymerization in the presence of cross-linking agents or initiators containing functional groups that react with the monomers, forming covalent bonds between polymer chains. Examples of polymerization methods include free radical polymerization, photopolymerization, and step-growth polymerization [24].

Another approach is cross-linking through chemical reactions, where pre-formed polymers or polymer precursors with functional groups react chemically to form cross-links. Functional groups on the polymers can react, forming covalent bonds and cross-linking the polymer chains. Common chemical reactions used include Michael addition, thiol-ene click chemistry, and Schiff base reactions [22,23,29].

Enzymes such as transglutaminase can also catalyze the formation of covalent bonds between proteins, leading to the cross-linking of polymer chains and the formation of hydrogels [25]. Certain hydrogels can undergo reversible cross-linking in response to physical stimuli like temperature, pH, or light [30,31]. Additionally, hydrogels can be cross-linked through intermolecular interactions, such as hydrogen bonding, electrostatic interactions, or host-guest interactions. These interactions can be reversible or irreversible, depending on the specific system [32].

The choice of method depends on various factors, including the desired properties of the hydrogel, the type of polymer or monomer used, and the specific application requirements. Each method offers different advantages and limitations in terms of control over cross-linking density, reaction kinetics, and the ability to incorporate bioactive molecules or functional groups into the hydrogel network.

## 3. Hydrogels Research in Italy

Italian researchers are actively exploring the use of hydrogels in areas including, but not limited to, tissue engineering, drug delivery systems, wound healing, regenerative medicine, and pathophysiological research.

### 3.1. Hydrogels Derived from ECM

This section highlights the advancements and contributions of Italian research in the field of extracellular matrix-based hydrogels. We have subdivided the discussion based on the type of tissue or organ from which the hydrogel is derived.

#### 3.1.1. Decellularized Colorectal Cancer Mucosa and Derived Hydrogels

dECM derived from human cancer biopsies holds great promise as a platform for developing 3D-bioactive models to be used in cancer research.

Colorectal cancer (CRC) typically arises from epithelial cells through well-defined pathogenic processes [33]. However, despite a shared genetic background, CRC exhibits diverse trajectories in terms of growth, aggressiveness, and prognosis, which have been linked to alterations in ECM components and their accumulation in the tumor microenvironment. These changes can either restrict tumor growth or promote progression with adverse outcomes, indicating the complex and pivotal influence of ECM on cancer development [34,35]. Biologically-derived matrices contain essential structural proteins and soluble factors derived from the tissue of origin, thus facilitating more reliable tissue reconstruction in the context of 3D tumor engineering [36].

**Figure 1:**
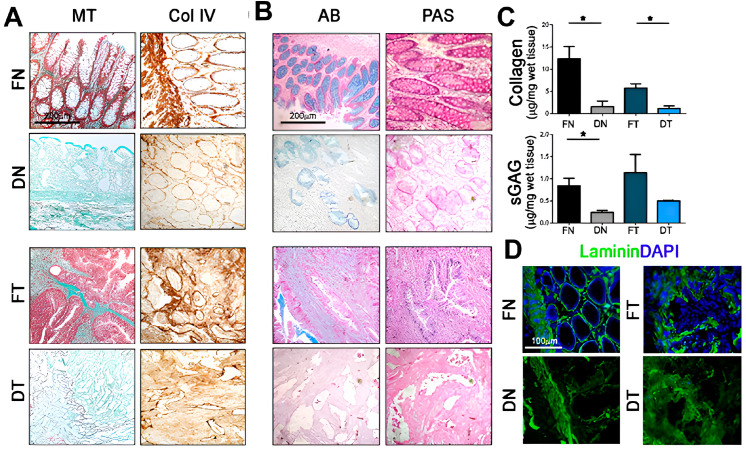
Decellularized tissue characterization. (**A**) Masson’s Trichrome (MT) and collagen IV (Col IV) stains for the detection of collagens in fresh (F) and decellularized (D) samples, both normal (N) and tumor-derived (T). (**B**) Alcian blue (AB) and periodic acid–Schiff (PAS) stains for the detection of polysaccharides, glycoproteins, and glycolipids in fresh and decellularized samples. (**C**) Quantification of collagens (I to V) and sulfated glycosaminoglycans (sGAG) in fresh and decellularized samples(*: *p*-value < 0.05). (**D**) Immunofluorescence of Laminin (green) in fresh and decellularized samples. Nuclei are counterstained with DAPI. (reproduced from [37] with permission from John Wiley & Sons, Hoboken, NJ, USA—Books).

Piccoli et al. employed this approach to create a specialized colorectal cancer (CRC) model that accurately replicates the native tissue’s ultrastructural characteristics. After two cycles of detergent-enzymatic treatment (DET cycles), they analyzed the composition of decellularized matrices using histology and protein quantification. While Masson’s Trichrome (MT) and Collagen IV stains indicated the presence and distribution pattern of collagens in both fresh and decellularized tissues, protein quantification revealed a significant decrease in total collagens in decellularized samples compared to their fresh counterparts (Figure 1A,C). Alcian blue (AB) and periodic acid–Schiff (PAS) stains confirmed the presence of GAG in both fresh (F) and decellularized tissues (D), but the quantification differed between normal (N) and tumor samples (T). Decellularized normal (DN) samples showed a significant decrease in protein quantity compared to the fresh ones (FN), while no significant differences were observed between decellularized (DT) and fresh tumors (FT) (Figure 1B,C). Immunostaining for laminin, another major ECM component, demonstrated its uniform distribution throughout the decellularized samples. This revealed the preservation of ordered physiological villi in DN samples and a disorganized, unstructured matrix in DT ones (Figure 1D). This clearly confirmed the success of their in vitro system in mimicking the native tissue’s properties. Comparisons between decellularized healthy mucosa and CRC through proteomic and secretomic analyses highlighted significant differences in the stromal composition. The biological properties of the 3D acellular matrices were preserved, and the decellularized CRC had reduced angiogenic potential compared to healthy tissue, primarily due to the involvement of Defensin Alpha 3 (DEFA3). Furthermore, matrices recellularized with HT29 cells over the course of 5 days showed an upregulation of IL-8, a chemokine involved in cancer growth and proliferation via a pathway mediated by DEFA3 [37].

D’Angelo et al. developed a 3D CRC model, along with colorectal liver metastasis (CRLM), using decellularized ECM scaffolds from patient samples. Following two DET cycles, both healthy liver (HL) and CRLM retained a phenotypical appearance similar to their pre-DET counterparts without evident degradation or disintegration. Analysis through scanning electron microscopy (SEM) and DAPI staining confirmed successful cellular depletion and preservation of the ultrastructure. Immunohistochemical analysis demonstrated that the expression and distribution of key ECM components, such as collagen and glycosaminoglycans, were maintained in post-DET HL and CRLM tissues. This approach provided further insight into the behavior of HT29 cells within cancer-derived scaffolds, resulting in increased proliferation and migration compared to healthy tissues. The model also showed traits of epithelial-mesenchymal transition (EMT), a hallmark of cancer progression. Interestingly, cells cultured in 3D cancer-mimicking conditions had a reduced sensitivity to conventional chemotherapy treatments with 5-fluorouracil and the combination of 5-fluorouracil with Irinotecan, compared to their two-dimensional counterparts. This patient-specific 3D model proved invaluable for studying the progression of CRLM and assessing the efficacy of chemotherapy agents [38].

Sensi et al. introduced a pre-clinical model for drug evaluation based on patient-derived 3D cultures. Surgically resected CRC tissue and the adjacent healthy colon mucosa were decellularized with two DET cycles and used to create an in vitro model representative of the patient’s disease. Following cell depletion, the ECM was again maintained, and the ultrastructure was preserved in both N and T samples. Re-cellularization with HT29 and HCT116 cells and exposure to 5-fluorouracil revealed reduced drug sensitivity compared to traditional 2D cultures. This model bridged the gap between traditional in vitro testing and in vivo drug efficacy, offering insights into patient-specific treatments [39].

Taken together, these studies prove the importance of considering and integrating bioactive components such as dECM matrices into CRC and cancer research in general. Turing native tissues into scaffolds for in vitro experiments brings our studies a step closer to the true behavior of living cells and tissues. In addition to the advances in knowledge, the overall goal is to develop improved and more effective therapeutic strategies.

#### 3.1.2. Hydrogels Derived from Decellularized Skeletal Muscle

Recent studies explored the potential of dECM derived from skeletal muscle for investigating the interactions between cells during myogenesis and exploring innovative approaches to address conditions like Duchenne Muscular Dystrophy (DMD) and volumetric muscle loss (VML).

Auletta et al. used decellularized skeletal muscle extracellular matrix (SkM ECM) to direct the differentiation of human induced pluripotent stem cells (hiPSC) into structured tissue-like neuromuscular organoids (t-NMOs). Despite complete cell removal required here in three DET cycles, the preservation of structural and topographical features of native tissue was still maintained. The decellularized SkMs preserved their mechanical properties, as verified via Atomic Force Microscopy analysis (AFM). Over the course of 30 days, the t-NMOs matured, exhibiting contractile activity and functional neuromuscular junctions (NMJs). Interestingly, the t-NMOs replicated altered muscle contractions caused by neurotoxins targeting NMJs, highlighting their accuracy in modeling physiological responses. Additionally, the model successfully recreated the abnormal contractility and calcium dynamics seen in DMD by generating DMD t-NMOs using hiPSCs from affected individuals [40].

**Figure 2:**
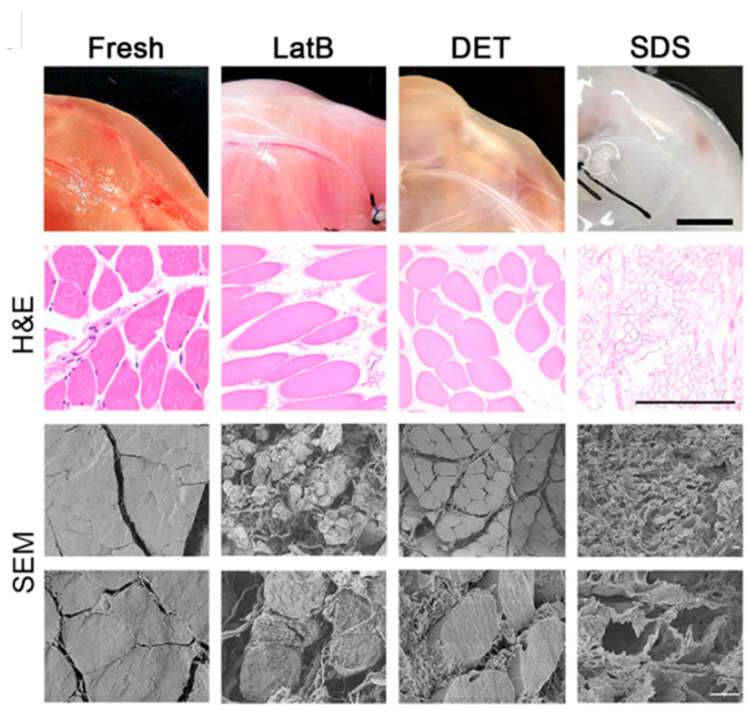
Macroscopic and microscopic (H&E and SEM) evaluation of fresh rat muscles and LatB-, DET- and SDS-decellularized samples. For H&E analysis, cross-sections were analyzed. Scale bars, macroscopic: 1 cm; H&E: 100 μm; SEM: 10 μm. (reproduced from [41] with permission from Springer Nature. CreativeCommons Attribution 4.0 International License).

Urciuolo et al. studied muscle regeneration using decellularized skeletal muscle as scaffolds. They achieved VML through surgical removal of the extensor digitorum longus (EDL) muscle in a xenogeneic immune-competent model. Decellularized scaffolds were generated using three distinct methods: LatB, DET, and SDS. LatB effectively removed the cellular components and preserved the ECM structure in a single cycle, while DET required three cycles and SDS needed 72 h of perfusion for equivalent decellularization levels. The treated tissues exhibited varying macroscopic appearances. In comparison to freshly isolated muscles, all decellularization protocols significantly reduced DNA content, as confirmed by both H&E staining and SEM, but revealed distinct structural characteristics. LatB and DET yielded samples with preserved general tissue structure, including myofibers. In contrast, SDS treatment resulted in a significant depletion of myofiber content, leaving mainly the ECM (Figure 2). All dECMs used as substitutes for the excised muscles facilitated host myogenic cell migration and differentiation, alongside the formation of nervous fibers and vascular networks and the recruitment of satellite cells (SCs). However, other scaffolds primarily composed of ECM outperformed those preserving native muscular cytoskeletal structures, showing superior myofiber organization and SC population restoration. Overall, the authors prove the importance of their models for understanding cell interactions during myogenesis and indicate the potential of xenogeneic acellular muscle in VML treatment [41].

Piccoli et al. have successfully shown, for the first time, that the orthotopic transplantation of a decellularized diaphragmatic muscle can facilitate tissue functional recovery in a mouse model of muscle atrophy. The ECM in the scaffold triggered a local immune response, creating a pro-regenerative environment and promoting the activation and migration of muscle progenitor cells in the host. These findings suggest that acellular scaffolds have the potential to serve as an effective regenerative medicine approach for diseased muscles. Diaphragms destined for the decellularization process were treated with one to four DET cycles. Immunofluorescence, H&E staining, immunohistochemistry, and nuclei quantification set three DET cycles as the necessary threshold [42].

Further studies on dECM from mice diaphragms by Trevisan et al. aimed at mimicking clinical requirements for off-the-shelf use in skeletal muscle reconstruction. Diaphragm muscles from 3-month-old mice were processed with three DET cycles to remove all cellular material. The dECMs were stored under different conditions prior to seeding with pediatric human muscle precursor cells to monitor proliferation and differentiation. The engineered 3D skeletal muscle constructs were then subjected to cardiotoxin injury, and they functionally responded by activating cells’ self-renewal and ECM remodeling. The successful reconstruction of functional skeletal muscle while maintaining a pool of stem cells demonstrated the potential of this approach for future clinical applications in diaphragmatic regeneration [43].

Boso et al. successfully developed and characterized a hydrogel derived from porcine diaphragms for diaphragmatic applications. Samples were processed with four DET cycles to obtain complete cell removal. After decellularization, matrices were washed for at least 3 days with deionized sterile water, lyophilized, and finally ground to fine powder. Lyophilized powder (at concentrations of 1%, 2%, and 3% *w*/*v*) underwent enzymatic digestion in pepsin and acetic acid. To restore the solution’s osmolarity and mimic physiological conditions, the pH was adjusted to 7.4, and 10% *v*/*v* 1× PBS was added. Gelation of the neutralized solution was initiated by maintaining it at 37 °C until collagen fibrils spontaneously formed, resulting in hydrogel formation. Cross-linking of the hydrogels was induced using genipin. The hydrogel exhibited a tissue-specific composition closely mimicking the complex structure of skeletal muscle ECM. They extensively characterized the properties of the hydrogel, including biomechanical properties, biocompatibility, and its suitability for in vivo applications. Their findings demonstrated that the dECM-derived hydrogel derived from porcine diaphragms could repair diaphragmatic muscle defects when used as an acellular patch, offering a promising approach for diaphragmatic muscle repair [21].

In summary, recent research involving decellularized skeletal muscle ECM showcases its ability to guide tissue development, facilitate muscle regeneration, and serve as a platform for disease modeling. These findings offer significant insights into the potential applications of dECM in addressing musculoskeletal conditions and advancing our understanding of cellular interactions in muscle development.

#### 3.1.3. Hydrogels Derived from Decellularized Pericardium

The pericardium is a double-layered sac surrounding the heart, providing protection and stability. Pericardium-derived dECM can thus serve as a natural scaffold for tissue repair and regeneration, with potential applications in cardiac and vascular therapies.

Di Francesco et al. analyzed the complex macromolecular composition of a hydrogel derived from dECM obtained from bovine pericardium, highlighting an abundance of collagen type I. The hydrogel was obtained with a procedure similar to the one used by Boso et al. [21]. The hydrogel exhibited excellent biocompatibility and induced the polarization of macrophages towards an M2 phenotype. In addition, it promoted the expression of angiogenic markers and maintained functionality in a hindlimb ischemia model. Furthermore, the hydrogel facilitated the migration of fibroblasts and showed promise in promoting wound closure in vivo [26].

Overall, this dECM hydrogel derived from bovine pericardium showed complex composition, excellent biocompatibility, immune modulation, angiogenesis, and wound healing promotion, all suggesting promising applications in tissue regeneration.

Table 1 summarizes the principal applications of the works regarding the use of hydrogels derived from ECM.

### 3.2. Non-ECM Hydrogels

This section highlights the advancements and contributions of Italian research in the field of non-extracellular matrix-based hydrogels in different areas of application.

#### 3.2.1. Drug Delivery Application

Hydrogels can also be key in the field of drug delivery, offering a unique combination of biocompatibility, tunable properties, and controlled release capabilities. High water content and tunable porosity, composition, cross-linking density, and degradation rates all allow for precise diffusion and control over drug release kinetics [46].

Malizos et al. presented initial clinical trial data involving a fast-resorbable hydrogel coating loaded with antibiotics (known as Defensive Antibacterial Coating or DAC^®^) to prevent surgical site infections in patients undergoing internal osteosynthesis for closed fractures. Coating the implants with this antibiotic-loaded hydrogel reduced post-surgical site infections with no adverse events or side effects [32].

Zoccali et al. further demonstrated that DAC^®^ is effective in significantly reducing surgical site infections in various clinical scenarios. Through a matched case-control design, they proved that the application of a fast-resorbable coating loaded with antibiotics is a safe approach for protecting joint mega-prostheses by reducing the incidence of early surgical site infections without any adverse effects [47].

Furlani et al. focused on creating an injectable nanocomposite for regenerative medicine applications. The system was based on a chitosan thermosensitive hydrogel combined with liposomes. Chitosan and β-glycerophosphate solutions were prepared separately. Chitosan was dissolved in deionized water containing 1% *v*/*v* acetic acid to a concentration of 25 mg/mL, while β-glycerophosphate was dissolved in deionized water to a concentration of 645 mg/mL. Following overnight refrigeration at 4 °C, the β-glycerophosphate solution was added dropwise to the chitosan solution in an ice bath under magnetic stirring. The resulting mixture was incubated overnight at 37 °C to facilitate hydrogel formation. The hybrid system was created by combining liposomes and a chitosan solution (dissolved in 1% *v*/*v* acetic acid) at 4 °C and a volume ratio of 1:4.56 (liposomes to chitosan). This nanocomposite hydrogel enabled the controlled release of the liposomal content, facilitating interactions with cells and internalization. The presence of a chitosan coating enhanced cellular uptake and internalization compared to cells treated with liposomes alone. The gelation temperature was 32.6 °C, allowing easy injection at the target site and hydrogel transition at body temperature. Due to its unique performance, this thermosensitive hydrogel holds great potential as a controlled delivery system for liposomes in tissue regeneration applications [48].

Laurano et al. created a novel thermo- and pH-responsive hydrogel (P-CHP407) using an amphiphilic poly(ether urethane) (CHP407) synthesized specifically for the controlled and localized release of drugs. The P-CHP407 hydrogel had a significant amount of exposed -COOH groups (8.8 ± 0.9 nmol/g_polymer_), the consequence of optimized plasma treatment on the powder, leading to a slightly lower initial gelation temperature (12.1 °C) compared to the CHP407 hydrogel (14.6 °C). Nanoscale characterization using Low Field NMR (LF-NMR) spectroscopy indicated that the P-CHP407 hydrogel formed larger micelles with a thicker hydrated shell than the CHP407 hydrogel, as further supported by Dynamic Light Scattering analyses. Moreover, the P-CHP407 hydrogel demonstrated an improved ability to change its internal pH when exposed to an alkaline buffer (pH 8) compared to the CHP407 hydrogel (e.g., pH change after 5 min is 3.76 vs. 1.32, respectively). LF-NMR analysis suggested stronger alkaline-pH-induced interactions of water molecules with the micelles containing -COOH groups in the P-CHP407 hydrogel. Both hydrogels were biocompatible according to ISO 10993 standards and capable of loading and releasing Ibuprofen [49]. The P-CHP407 hydrogel exhibited enhanced Ibuprofen delivery kinetics at alkaline pH, indicating its potential use as a smart delivery system for the treatment of chronically infected wounds and wound healing applications [50].

High doses of the antitumoral drug doxorubicin (Dox) are associated with cardiotoxic side effects and myelosuppression. In this context, Gallo et al. developed hydrogels (HGs) and nanogels (NGs) using short peptide sequences to deliver Dox or its liposomal formulation, Doxil. HGs and NGs formulations were based on the use of Fmoc-FF alone or in combination with (FY)3 or PEG8-(FY)3 peptides at different ratios (1/1 and 2/1 *v*/*v*). HGs were created using the “solvent-switch” method, while NGs were obtained by the submicronition of the HGs with TWEEN^®^60 and SPAN^®^60 as stabilizing agents. In vitro assays were performed on MDA-MB-231 breast cancer cells using both empty and Dox-filled HGs and NGs. When examining drug internalization by cells (via immunofluorescence assays), the authors proved that Dox-filled hydrogels showed a high drug-loading content (DLC = 0.440) and were stable without any syneresis for 10 days. The gelation kinetics (20–40 min) and drug release over time (16–28%) of the HGs were influenced by the relative peptide composition. The Dox-filled NGs had a DLC of 0.137 and exhibited a low drug release (20–40%) after 72 h. Empty HGs and NGs had high cell viability (>95%), while Dox-loaded ones significantly reduced it after 24 (49–57%) and 72 h (7–25%) of incubation, respectively. Immunofluorescence assays showed distinct cell localization patterns for Dox delivered through HGs and NGs compared to the free drug, suggesting alternative internalization mechanisms. By adjusting the ratios of the peptide components, they could also modulate Dox release from the HGs. Overall, the high drug loading capacity, controlled release, and promising in vitro results suggest the potential use of peptide-based HGs and NGs for drug delivery applications [29].

Parisi et al. introduced a drug delivery system utilizing a heterochiral tripeptide hydrogel (D-Leucyl-phenylalanyl-phenylalanine (D-Leu-Phe-Phe)) to embed the antineoplastic drug, 5-fluorouracil (5-FU). The peptide was initially dissolved in sodium phosphate buffer (0.1 M, pH 12.0), followed by the addition of an equal volume of sodium phosphate at acidic pH (0.1 M, pH 5.8) to achieve a peptide concentration of 10 mM and a pH of 7.2 ± 0.1. Next, the peptide was dissolved in sodium phosphate buffer (0.1 M, pH 11.8), to which a specific amount (5% of the final volume of the hydrogel) of 5-fluorouracil in 1 M NaOH was added. Finally, half of the final volume of the hydrogel of sodium phosphate monobasic dihydrate (0.1 M, pH 4.5) was added to adjust the pH to 7.3 ± 0.1. The final concentrations of the peptide D-Leu-Phe-Phe and 5-fluorouracil were 10 mM and 13 mM, respectively. They demonstrated that antitumoral drugs can be effectively incorporated into a supramolecular hydrogel primarily composed of a tripeptide, even at clinically relevant concentrations. Despite 5-FU being hydrophilic, its aromatic nature and hydrogen bonding potential enable interactions with the peptide matrix. However, experiments revealed that 5-FU was rapidly released within a few hours, indicating a lack of strong interactions with the peptide assemblies. The study suggests that exploring other structural features of drug molecules may lead to the development of supramolecular hydrogels capable of slower and controlled drug release profiles over several days, holding significant implications for the design of drug delivery systems with enhanced therapeutic efficacy [24].

Ciocci et al. incorporated protein microspheres (MS) into a composite biosynthetic hydrogel to enhance porosity and cell-adhesive properties. The backbone of the gel contained silk fibroin (SF) for its bio-functionality and polyethylene glycol diacrylate (PEGDa) for its structural versatility. The addition of chondroitin sulfate also improved cell viability. Cardiac mesenchymal stem cells cultured within the MS-embedded hydrogel were viable and expressed proteins characteristic of the early stages of cardiac muscle differentiation. Overall, their results indicated that hydrogel functionalized with MS can serve as stem cell-carriers with sponge-like properties, making them suitable for applications as ultrasound-imaging contrast agents and controlled delivery of biochemical factors [22].

#### 3.2.2. 3D Bioprinting Application

The advent of 3D bioprinting put hydrogel at the core of the development of new and improved bioinks for the generation of tissue constructs for applications ranging from basic discovery to tissue engineering and regenerative medicine.

Cochis et al. developed a 3D printing method using extrusion bioprinting of methylcellulose (MC)-based hydrogels. MC hydrogels were formulated using a dispersion technique. Aqueous solutions were prepared using either a 0.05 M Na_2_SO_4_ or a 20 g/L phosphate-buffered saline (PBS) solution. MC powder was added to the heated saline solutions at 55 °C to ensure uniform dispersion of the powder. The resulting polymer suspensions were then refrigerated at 4 °C for 24 h to achieve complete hydration of the methylcellulose. In vitro validations were performed using murine embryonic fibroblasts (NIH/3T3) and endothelial murine cells (MS1). Cell sheets growing on ring-shaped printed hydrogels oriented within the rings and had elongated nuclei compared to sheets from bulk hydrogels. The possibility of precisely aligning cells holds promise for tissue regeneration applications, especially in complex tissue engineering scenarios [30].

Magli et al. proposed the development of 3D printable hydrogels by cross-linking chitosan and gelatin, both functionalized with methyl furan groups. The reductive amination with methyl furfural, involving the lysine residues of gelatin and the amino groups of chitosan, resulted in hydrogels with customizable properties. The methyl furan residues led to cross-linking through Diels–Alder ligation with Star-PEG-maleimide under conditions that are compatible with cells. The chitosan-gelatin hybrid was used to formulate 3D printable hydrogels with good processability and biocompatibility [23]. This bioink was further perfected by Loi et al., who standardized the hydrogel formulation and its reproducibility. They produced high-resolution grids starting from 4 h after the addition of a cross-linker and succeeded in culturing rat osteosarcoma cells (UMR-106) for up to 14 days. After that time point there was evidence of construct fragmentation, likely due to interactions with the osteosarcoma cells [51].

Scarpa et al. optimized the fabrication of high molecular weight (10 kDa) PEGDa-based formulations for pH sensing in soft biological tissues. Pre-polymer solutions were created by dissolving PEGDa in MilliQ water and 0.67% *w*/*w* of the photoinitiator Irgacure™ 2959. 15 min stirring at 20 °C ensured perfect mixing. The solutions were sandwiched between two quartz glass slides separated by a poly(dimethyl) siloxane (PDMS) chamber and polymerized with a 10 min UV light exposure. Using two-photon lithography (2PL), they created microstructures shaped like pyramids and domes. These microstructures were then tested for mechanical properties and proved their ability to respond to changes in pH at the microscale by rapid swelling (less than 15 min). This development has promising implications for creating pH-sensitive materials for use in various biological and biomedical applications [11].

**Figure 3:**
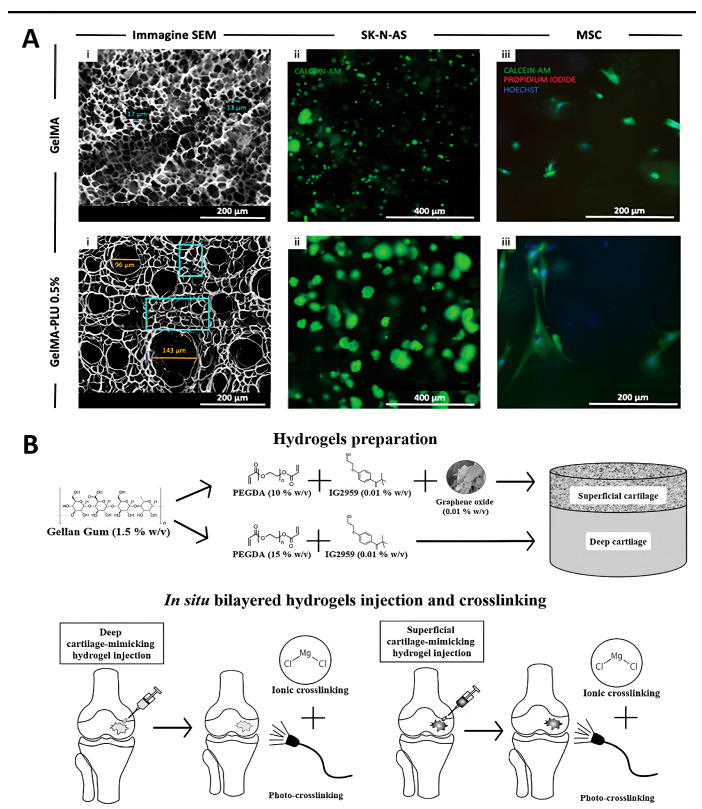
(**A**) Cancer cells organization within 3D constructs. Embedded cells, after 5 days of culture, show material-dependent behaviors. Top row: Pure GelMA: (i) SEM image showing representative pore diameters highlighted in blue; (ii) SK-N-AS cancer cells (green: Calcein AM staining the cytoplasm of live cells) are mostly isolated or in small aggregates; (iii) MSCs are similarly isolated and round. Bottom row: GelMA-PLU 0.5%: (i) SEM image: areas filled with microscopic pores (turquoise) as in pure GelMA, and mesoscale pores (yellow); (ii) live SK-N-AS cells (green: Calcein AM) embedded in GelMA-PLU 0.5% aggregate in large clusters, (iii) MSCs elongate and stretch. Scale bars as indicated. (reproduced from [10] with permission from John Wiley & Sons—Books) (**B**) Combinations of GG, PEGDA, and GO allow mimicking the properties of superficial and deep cartilage, respectively. Superficial and deep cartilage-mimicking hydrogels could be injected in situ and UV-crosslinked (photo-crosslinking) and/or polymerized with a CaCl_2_ solution (ionic cross-linking). (reproduced from [52] with permission from John Wiley & Sons—Books).

In Bova et al., we see the tuning of a bioink internal porosity by blending GelMa with Pluronic F-127 (PLU) (Figure 3A(i)). PLU-generated mesoscale pores resulted in increased swelling and a slight decrease in the Young’s modulus. All blends formed stable hydrogels, both when cast in annular molds and 3D bioprinted into complex structures. Embedded cancerous (Neuroblastoma, SK-N-AS cell line) (Figure 3A(ii)) and healthy Mesenchymal Stem Cells (MSCs) (Figure 3A(iii)) maintained high viability and physiologically correct behaviors dictated by the control over the micro- and macro-architecture of the constructs [10].

This relatively young field offers a unique combination of tunable materials, complexity in fabricated structures, and parameter control, giving it a central role in several applications. The technology supporting the design and production of Bioprinters is also advancing at a fast pace and providing ever-improved capabilities (i.e., coaxial printing). The cited works thus represent just a few recent examples and should serve as a reference proving the importance of hydrogel research in 3D bioprinting.

#### 3.2.3. Regenerative Medicine Application

Hydrogels can be tailored to mimic biological tissues thanks to their tunable properties, biocompatibility, and ability to encapsulate cells and bioactive molecules, making them a viable option for promoting tissue regeneration and repairing damaged organs. Researchers can adjust parameters such as cross-linking density, composition, and degradation rates to create hydrogels with desired properties suitable for various tissue types.

Trucco et al. used combinations of materials to mimic the zonal architecture of human articular cartilage (AC), proposing a two-layered structure composed of 1.5% *w*/*v* gellan gum (GG) and varying quantities of poly (ethylene glycol) diacrylate (PEGDa) (Figure 3B). The first combination, called “SUP”, consisted of PEGDa at 10% *w*/*v* and GG to simulate the superior cartilage layer. The second, “DEEP”, included PEGDa at 15% *w*/*v* and GG to represent the deep cartilage layer. The composite hydrogel had excellent resistance to wear, as validated through knee simulator tests and good biocompatibility following in vitro experiments with human chondrocytes [52]. Given its promise as a synthetic material for AC defect repair, Affatato et al. further studied its wear behavior in a four-station displacement control knee joint simulator. Roughness and micro-computer tomography (μ-CT) measurements highlighted a better performance of 15% *w*/*v* of PEGDa gels compared with 10% *w*/*v* PEGDa ones, which had higher roughness and less uniform density. The addition of graphene oxide (GO) helped maintain the hydrogel construct’s properties. The degree of cross-linking increased along the series: SUP < DEEP + SUP < DEEP without GO. Raman spectroscopy revealed the presence of unreacted C=C bonds in all hydrogels and the loss of diacrylate groups due to the washout of unreacted PEGDa in the bovine calf serum environment. This loss decreased along the series SUP > DEEP + SUP > DEEP, emphasizing the importance of the degree of photo-crosslinking in determining the wear behavior of the hydrogels [27].

Fuoco et al. used polyethylene glycol-fibrinogen (PF)-based materials to rejuvenate adult skeletal muscle-derived pericytes (MP). PF was prepared at the desired concentration and then diluted with sterile PBS as needed. A photoinitiator (Irgacure™ 2959) was incorporated into the PF mixture at a final concentration of 0.1% *w*/*v*. Cells were introduced at the desired concentration, and the solution was promptly exposed to UV light. They proved that adult MPs cultured in PF-based hydrogel scaffolds significantly improved their differentiation into muscle cells and angiogenic (blood vessel-forming) potential both in vitro and in vivo. Therefore, PEG-based hydrogel scaffolds offer a conducive environment or “niche” for progenitor cells, promoting skeletal muscle regeneration and blood vessel growth [53].

Vannozzi et al. developed a unique implantable muscle construct using programmed self-folding of PEGDa hydrogels. The construct consisted of two layers with different swelling degrees, stiffnesses, and thicknesses, capable of folding into a 3D tube-like structure. PEGDa solutions were formulated by dissolving PEGDa at a concentration of 20 wt% in phosphate-buffered saline (PBS). Irgacure™ 2959 was incorporated into each PEGDa solution at a concentration of 0.5 wt% to facilitate cross-linking upon exposure to UV light. Gelatin derived from porcine skin, specifically type A, was employed as a sacrificial thermoresponsive layer to initiate the detachment of the bilayer structure. Both skeletal and cardiac muscle cells were seeded in the construct, maintaining high viability, proper adhesion, and correct alignment. Remarkably, cardiac myocytes retained their contractility for up to 7 days. Biocompatible shape-changing materials open exciting possibilities for creating new cellular constructs for hierarchical tissue assembly, drug testing platforms, and biohybrid actuators capable of performing sophisticated tasks [54].

With an interest in bone tissue regeneration, Fiorati et al. developed an injectable hydrogel containing TEMPO-oxidized cellulose nanofibers (TOCNFs) embedded with inorganic calcium phosphate (CaP) and calcium phosphate-graphene oxide (CaPGO) particles. These inclusions served as physical cross-linkers and improved mechanical properties. However, rheological injection tests measured average load values in the 1.5–4.4 N range, well below the upper limit of 30 N, which is considered an acceptable injection force. Samples were stable over a 28-day period, and both CaP and CaPGO facilitated mineralization, as evidenced by SEM and XRD analyses. Furthermore, eluate tests on SAOS-2 cells demonstrated no cytotoxic effects. This study successfully demonstrates that the addition of inorganic phases to TOCNFs-based dispersions enhanced and modulated their physicochemical properties while preserving injectability and bioactivity [28].

Alessio et al. investigated the impact of hyaluronic acid (HA), chondroitin sulfate (CS), and bio-fermentative unsulphated chondroitin (BC) on the commitment and differentiation of mesenchymal stem cells (MSCs) into chondrocytes in vitro. These glycosaminoglycans (GAGs) were added in combination and at different stages of the cultivation process. The main findings revealed that supplementing GAGs during the terminal phase of in vitro differentiation significantly improved chondrocyte maturation without causing fibrosis (reduced expression of Type I collagen). In summary, the study emphasizes the importance of carefully considering in vivo-like extracellular cues during cell differentiation and maturation for an optimized tissue engineering approach [55].

Regenerative medicine is probably one of the most popular applications for hydrogel-derived biomaterials, represented and proved by the large numbers of published papers and, most importantly, several products reaching the clinic and the market.

#### 3.2.4. Pathophysiology In Vitro Models

Dupont et al. studied mechanotransduction by monitoring the transcriptional activity of YAP/TAZ in human mammary epithelial cells (MEC) cultured on fibronectin-coated acrylamide hydrogels (FCAh) with varying stiffness. YAP (Yes-associated protein) and TAZ (transcriptional coactivator with PDZ-binding motif) are transcriptional coactivators and key components in mechanotransduction, thus capable of responding to mechanical cues from the extracellular environment to then influence cellular behavior. These hydrogels were designed to match the physiological elasticities of natural tissues, with elastic modulus ranging from 0.7 to 40 kPa. The results showed that cells grown on stiff hydrogels (15–40 kPa) exhibited YAP/TAZ activity levels like cells grown on conventional plastic surfaces. On the other hand, cells on soft matrices (in the range of 0.7–1 kPa) recorded inhibited YAP/TAZ activity, comparable to cells where YAP/TAZ was depleted using short interfering RNA (siRNA). These findings prove that the mechanical properties of the ECM play a crucial role in regulating YAP/TAZ activity in mammary epithelial cells [56].

Serena et al. similarly proved the fundamental role of substrate mechanical properties in an in vitro dystrophy model. The researchers produced thin films of poly-acrylamide hydrogels (Pah) with tunable mechanical properties (elastic moduli, E: 12, 15, 18, and 21 kPa) and micro-patterned different adhesion proteins (laminin, fibronectin, and matrigel) in parallel lanes (75 mm wide, 100 mm spaced) to maximize myoblasts alignment and fusion. Briefly, the hydrogels were polymerized through activation of Irgacure™ 2959 via UV light exposure. Selective photopolymerization was achieved by positioning a photomask with the desired geometry between the light source and the solution. Human healthy and dystrophic myoblasts cultured on the hydrogels differentiated into myotubes with functional sarcomere formation. The highest percentage of myotubes (60.0% ± 3.8) exhibiting organized myosin heavy chain II and α-actinin was measured after 7 days of culture on an elastic (15 kPa) hydrogel with matrigel patterning. Furthermore, healthy myotubes cultured in these conditions demonstrated significant membrane-localized dystrophin expression. This approach provided a versatile platform for a wide range of studies on human muscle physiopathology [9].

Cavo et al. identified a specific gel composition (50% Alginate, 50% Matrigel) inducing physiological behaviors in human breast cancer cells (MDA-MB-231) cultured in three dimensions. The hydrogels were stable over time, biologically active, and compatible with their innovative bioreactor-based invasion assay. Through a detailed morphological characterization, the authors proved that cancer cells exhibited distinct cytoskeleton shapes and nuclear fragmentation, which are characteristic features of their malignancy, and formed invadopodia, actin-based protrusions of the plasma membrane that allow cells to anchor to the extracellular matrix. Cells also migrated through the gels and attached to an engineered membrane mimicking vascular walls within the bioreactor. Overall, the study successfully developed a biologically active 3D substrate for breast cancer cell culture, enabling the observation and analysis of crucial steps in the metastatic process [57].

These studies collectively exemplify that exploiting hydrogels, combining their capacity to create a biologically relevant microenvironment for cultured cells with fine-tuning of key parameters such as stiffness and composition, can provide improved platforms to study several mechanisms of human diseases.

### 3.3. Hydrogels and Bioreactors

To further improve our capacity to in vitro mimic in vivo processes, it is necessary to incorporate some forms of perfusion and/or recirculation of fluids. Engineered systems such as bioreactors and micro-bioreactors, categorized based on the volumes of fluids at play, are the most common tools performing the above-mentioned tasks. Reactors are also designed to provide additional biophysical stimuli, such as mechanical or electrical, to cells and tissues in culture [58,59]. Of relevance to our review, micro-bioreactors (or microfluidic devices) are compatible with the culture of 3D hydrogel-based cellular constructs, thus combining all the above-mentioned properties and advantages of hydrogels with the unparalleled control over culture parameters of microscale technologies [60].

#### 3.3.1. Regenerative Medicine Application

Todros et al. designed a novel bioreactor to stimulate porcine-derived diaphragmatic scaffolds with the goal of creating clinically relevant tissue patches. Diaphragms were processed with 3 DET cycles in order to obtain a complete cell removal. Tensile tests were performed on both fresh and decellularized samples of porcine diaphragmatic tissue to understand their mechanical behavior and feed the Finite Element (FE) model, aiding design optimization for the bioreactor. Numerical analyses were then conducted by applying pressure to the bioreactor membrane and evaluating tissue strain during stimulation. The bioreactor was then designed to allow radial mechanical stimulation of tissue patches by uniformly applying up to 30% strain while avoiding tissue laceration [44]. The same bioreactor was also used by Maghin et al., who used decellularized diaphragmatic muscle and human cells to create diaphragmatic-like tissues in vitro. The aim was to provide a new option for surgically treating large diaphragm defects. In vitro tests showed that the bioreactor culture positively influenced ECM remodeling and fibroblast overgrowth. The mechanically stimulated constructs exhibited increased tissue maturation, with the development of new, oriented, and aligned muscle fibers. Furthermore, when implanted into a surgical congenital diaphragmatic hernia (CDH) mouse model, the mechanically stimulated tissues sustained human cells within the myofibers with no hernia recurrence [45].

Testa et al. proposed a novel approach for tendon tissue engineering. They exposed C3H10T1/2 murine fibroblasts to a combination of conditions comprising a biochemical stimulus using Transforming Growth Factor Beta (TGF-b) and Ascorbic Acid (AA); a 3D environment using PF as a biomimetic matrix; a mechanical induction through a custom bioreactor applying uniaxial stretching. Immunofluorescence and mechanical testing in vitro proved the enhanced mechanical strength of the engineered tissue and a remarkable 3D arrangement of cells and deposition of neo-extracellular matrix. The combined effect of biochemical and mechanical stimuli greatly improved the biological and mechanical properties of the artificial tissue compared to single exposures [61].

Cochis et al. tested a novel thermoreversible hydrogel composed of 8% *w*/*v* methylcellulose (MC) in a 0.05 M Na_2_SO_4_ solution to improve chondrogenesis. The MC hydrogel was prepared using a dispersion technique and thoroughly characterized. The hydrogel underwent a solution-gelation transition between 34 and 37 °C and exhibited less than 20% degradation after 1 month. The hydrogel showed no immunoreactivity or adverse reactions following subcutaneous implantation in mice, indicating its safety for potential biomedical applications. For inducing chondrogenesis in vitro, mesenchymal stem cells (MSCs) were seeded into the MC solution retained within a porous polyurethane (PU) matrix. These PU-MC composites underwent a combination of compression and shear forces in a custom-made bioreactor for 21 days. The mechanical stimulation resulted in a significant increase in chondrogenic gene expression, and histological analysis revealed the presence of sulfated glycosaminoglycans and collagen II only in the loaded specimens, confirming the ability of the MC hydrogel to support load-induced MSCs chondrogenesis [31].

#### 3.3.2. Pathophysiology In Vitro Models

**Figure 4:**
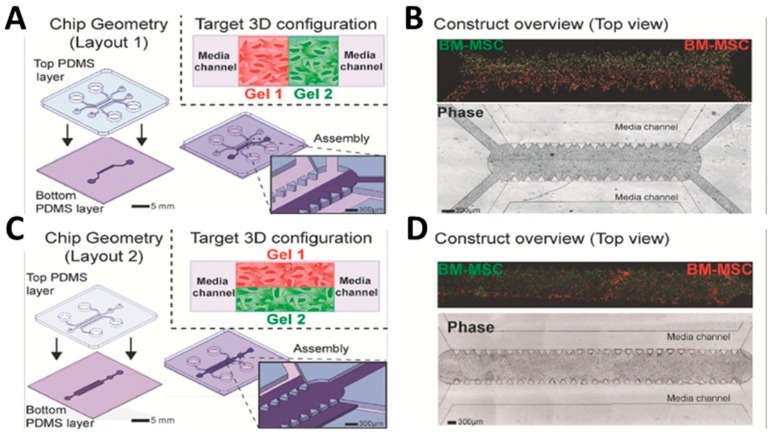
(**A**) Assembly of elastomeric layers forming flanked composites (Layout 1). The inset shows the desired configuration with two side-by-side hydrogels. (**B**) Overview of the entire 3D composite construct (at day 3) both in bright field and fluorescent imaging showing two cell-laden fibrin hydrogels (red and green). (**C**) Assembly of microdevice layers to form stacked composites (Layout 2). The inset shows the final construct configuration with two stacked hydrogels. (**D**) Overview of the entire stacked composite 3D construct (at day 3) both in bright field and fluorescent imaging showing two cell-laden fibrin hydrogels (red and green) (reproduced from [60] with permission from John Wiley & Sons—Books).

Ugolini et al. introduced a new method for shaping composite 3D cellular structures within microfluidic channels. This technique was based on the use of removable polydimethylsiloxane (PDMS) molds, enabling the gradual creation of composite 3D structures that could selectively integrate different cell types and/or biomaterials with precise spatial control. By using a combination of fibrin and collagen hydrogels, the method allowed the formation of either layered structures with seamless planar interfaces (Figure 4A,B) or parallel arrangements of hydrogels with vertical interfaces (Figure 4C,D). Fibrin gels were created by combining fibrinogen and thrombin to a final concentration of 20 mg mL^−1^ fibrinogen and 2 U mL^−1^ thrombin. Rat tail type I collagen was used to produce collagen gels with a final concentration of 3 mg mL^−1^. Cell-containing hydrogels were introduced into the gel inlets of the microdevices and allowed to undergo cross-linking within humidified chambers in standard cell culture incubators. Additionally, they demonstrated how this method can be used to produce customized endothelial barriers and single layers directly connected to 3D cellular structures [60].

Orsi et al. developed a novel device creating 3D concentration gradients of soluble species to be used for the differential perfusion of scaffolds while also enabling the fabrication of hydrogels with 3D gradients of mechanical properties. Polyacrylamide (PAAM) was chosen as a template hydrogel because of the ease in tuning its mechanical properties by just changing the acrylamide (AAM)/bisacrylamide (BIS) ratio (monomer/cross-linker ratio). The authors aided the design of the gradient generator via computational finite element analysis and validated the obtainment of the desired 3D gradient of stiffness through experimental studies. This device allows for simultaneous exposure to gradients in both soluble chemical species and substrate stiffness, opening unexplored studies of cell behaviors related to chemotaxis and mechanotaxis [62].

When building in vitro models for human tissues, it is important to consider the presence and role of the vasculature component. Arrigoni et al. studied the ideal configuration of vessel-like structures within fibrin gels. Aided by computational modeling, they devised a non-planar arrangement of at least three microchannels with a diameter of 600 μm to guarantee effective oxygen distribution throughout thick cylindrical hydrogels. Co-culturing mesenchymal and endothelial cells supplemented with ANG-1 and VEGF led to the development of a robust vascular network. The study also highlighted how the appropriate combination of bioreactor-induced shear forces and oxygen levels was heavily involved in the sprouting of microvessels and proper network formation. In particular, an oscillatory flow and the absence of hypoxic areas (known inducers of angiogenesis) were correlated to poor vessel outgrowth [63].

When working with cardiac tissue, an additional fundamental component is the constant presence of stretching and mechanical forces, all actively determining tissue development and maturation in vivo. Massai et al. designed an automated system enabling adjustable cyclic stretching and real-time monitoring of the mechanical behavior of in vitro-engineered cardiac tissues. 3D annular fibrin hydrogels were loaded with neonatal rat cardiac cells and subjected to combinations of uniaxial cyclic stretching (sinusoidal waveform, with a 10% strain at a frequency of 1 Hz) within a bioreactor. The results proved that cyclic stretching promoted the alignment, preservation, and maturation of cardiomyocytes compared to static controls. The bioreactor allowed for live monitoring, which revealed a gradual rise in the passive force generated by the constructs during dynamic culture. Notably, only the stretched constructs could be paced by external electrical stimulation to a synchronized and regular contractile activity [64].

Similarly, Bono et al. proposed a bioreactor compatible with the formation (“construct mode”) and stimulation (“culture mode”) of collagen-based tubular structures. The gel constructs contained smooth muscle cells (SMCs) and could be molded directly inside the bioreactor culture chamber and cultured for 5 days under 10% cyclic strain at a frequency of 0.5 Hz. Results revealed that cyclic stimulation led to a bi-axial compaction of the collagen matrix and a consequent enhancement in the mechanical strength of the strained samples compared to static controls. Additionally, cyclic strain led to a uniform distribution of cells throughout the entire thickness of the construct and maintenance of their contractility. In contrast, static cultures resulted in a reduction in overall cell density and uneven distribution with concentration at the outer rim of the constructs. In summary, this novel dual-mode bioreactor with engineered collagen-gel-based tubular constructs highlighted the importance of mechanical conditioning during in vitro maturation of muscle tissues [65].

Marasso et al. introduced a microfluidic chip designed to analyze cell movement within a 3D gel matrix. The PDMS device was replica molded from a standard silicon/SU-8 master and comprised a central microchannel flanked by micropillars and two reservoirs. The chip, compatible with high-resolution live microscopy, allowed studying both individual cells and 3D spheroids in response to precisely controlled chemical cues in space and time. The gel (Matrigel™) was confined in the cell culture volume, where the device succeeded in establishing a stable gel-liquid boundary and generating a diffusive chemoattractant gradient [66].

#### 3.3.3. Therapeutic Application

Lev et al. optimized the growth and culture of recombinant Cripto-1 protein-expressing HEK293 cells using a 3D hydrogel microcarrier. Cripto-1 is a biotherapeutic soluble protein that is recombinantly expressed in mammalian cells. The microcarriers were fabricated from poly(ethylene glycol)-fibrinogen (PF) hydrogels and designed to withstand suspension culture in stirred bioreactors for up to 21 days without deteriorating or degrading. PEGylated fibrinogen was prepared by conjugating PEGDa via Michael-type addition with denatured, reduced fibrinogen chains. The PF gels created a suitable environment for cell growth and protein production, resulting in significantly higher purified Cripto-1 yields compared to traditional two-dimensional (2D) culture systems. The Cripto-1 produced by cells grown on the 3D microcarriers had similar bioactivity to commercially available Cripto-1, demonstrating a promising potential for hydrogel-based approaches in improving biomanufacturing processes for therapeutic proteins [67].

#### 3.3.4. Biodetection

Another interesting application for hydrogel materials is in the field of biodetection. De Masi et al. created an on-chip sandwich immunoassay with cleavable PEG-based microparticles tailored with monoclonal antibodies to detect human immunoglobulin G in biological fluids. Precise manipulation of particle numbers led to improved specificity and sensitivity, reaching as low as 3 pM in both serum and urine samples. Hydrogel particles were integrated into a microfluidic device controlling fluid exchange, incubation, and washing for on-chip target detection (HyPoC). The HyPoC dramatically reduced the incubation time from 180 to just one minute and the washing volumes from 3.5 mL to 90 μL. On average, the dynamic detection range on-chip was from 0.07 to 1 nM, demonstrating the successful development of a versatile, rapid, and user-friendly point-of-care testing platform for immunoassays [68].

Table 2 summarizes the principal applications of the works regarding the use of hydrogels not derived from ECM in the field of biomedical applications.

## 4. Challenges and Limitations

Despite all the advantages and favorable properties of hydrogel-based systems presented in the previous sections, some difficulties and limitations should be mentioned. First of all, the choice of the ideal material for a defined application is not trivial. Each target tissue and/or cell type dictates specific “selection rules” based on adhesive cues, mechanical compliance, and 3D structure, to name just a few. Once the selection has been made, careful experimental planning is also advised to screen for the optimal concentrations of polymers in the solution as well as the degrees of functionalization and cross-linking. If UV light is required for polymerization of cellularized constructs, doses and exposure times should also be carefully selected. Another possible challenge, especially when working with hydrogels derived from the ECM and with other natural polymers, is their degradation. For in vitro experiments, materials should ensure the maintenance of the desired properties and integrity for the entire duration of the campaign, also following media changes or perfused culture. If implanted in vivo, they should ideally guide and promote native tissue regeneration and degrade at a rate complying with new matrix deposition. Sterilization methods should also be carefully evaluated, as they could interfere with and modify the properties of the materials. Finally, hydrogel synthesis and production are typically labor-intensive procedures involving week-long protocols, thus being difficult to scale up and standardize.

## 5. Conclusions and Future Directions

In conclusion, this manuscript attempted to provide a comprehensive, although not exhaustive, review of key studies in the field of hydrogel research for biomedical applications led by the Italian scientific community. We summarized some fundamental characteristics of hydrogels, including their classification, physical and chemical properties, as well as various methods of synthesis. Hydrogels are now at the forefront in advancing biomaterials science and tissue engineering, wide areas of research to which all the cited studies brought significant contributions.

Biomimetic materials derived from native tissues are key in recapitulating in vivo microenvironments and greatly advancing the fields of regenerative medicine and modeling of human diseases. Other natural and synthetic hydrogels can be tailored for applications in drug delivery, 3D bioprinting, and the study of physiological and pathological processes. By merging hydrogel engineering and bioreactor technology, the degree of control over the culture microenvironments further increases. The possibility of obtaining ever-improving mimics of in vivo components paves the way for great advancements in disease modeling and in the understanding of tissue physiology.

Overall, this manuscript reviews the active role of Italian scientists in the field of hydrogel research, with the aim to further possible collaborations and concerted efforts in such an interdisciplinary and challenging field.

## Figures and Tables

**Table 1:** Summary of recent Italian studies on dECM and dECM-derived hydrogels.

Tissue	Derivation	Application	References
	DET cycles	Clinical screening	Piccoli et al. [37]
CRC	DET cycles	Drug screening	D’Angelo et al. [38]
	DET cycles	Drug screening	Sensi et al. [39]
	DET cycles	In vitro pathophysiology	Auletta et al. [40]
	LatB/DET/SDS	In vitro pathophysiology	Urciuolo et al. [41]
SkM	DET cycles	Regenerative medicine	Piccoli et al. [42]
	DET cycles	Regenerative medicine	Trevisan et al. [43]
	DET cycles, L, P, D, N, G	Regenerative medicine	Boso et al. [21]
	DET cycles	Regenerative medicine	Todros et al. [44]
	DET cycles	Regenerative medicine	Maghin et al. [45]
PC	DET cycles, L, P, D, N, G	Wound healing/Regenerative medicine	Di Francesco et al. [26]

**Legend: CRC:** colorectal cancer; **DET:** detergent-enzymatic treatment; **SkM:** skeletal muscle; **LatB:** latrunculin B; **SDS:** sodium dodecyl sulfate; **L:** lyophilization; **P:** pulverization; **D:** digestion; **N:** neutralization; **G:** gelation; **PC:** pericardium.

**Table 2:** Summary of recent Italian studies on natural, synthetic, or hybrid hydrogels for biomedical applications.

Hydrogel	Origin	Application	References
DAC^®^	Hybrid	Drug delivery	Malizos et al. [32]
			Zoccali et al. [47]
Chitosan/β-glycerophosphate	Hybrid	Drug delivery/Regenerative medicine	Furlani et al. [48]
P-CHP407	Synthetic	Drug delivery/Wound healing	Laurano et al. [50]
Fmoc-FF/(FY)3 Fmoc-FF/PEG_8_-(FY)3	Synthetic	Drug delivery	Gallo et al. [29]
D-Leu-Phe-Phe	Synthetic	Drug delivery	Parisi et al. [24]
SF/PEGDa	Hybrid	Drug delivery	Ciocci et al. [22]
MC	Hybrid	3D bioprinting/TE	Cochis et al. [30]
GE-MF:CH-MF:Star-PEG-MA	Hybrid	3D bioprinting	Magli et al. [23]
			Loi et al. [51]
		3D bioprinting (2 PL)	Scarpa et al. [11]
PEGDa	Synthetic	Regenerative medicine	Vannozzi et al. [54]
		Biodetection	De Masi et al. [68]
GelMA/PLU	Hybrid	3D bioprinting	Bova et al. [10]
PEGDa/GG/GO	Hybrid	Regenerative medicine	Trucco et al. [52]
			Affatato et al. [27]
		Regenerative medicine	Fuoco et al. [53]
PF	Hybrid	Regenerative medicine	Testa et al. [61]
		Drug delivery/Therapeutics	Lev et al. [67]
TOCNFs/CaP	Hybrid	Regenerative medicine	Fiorati et al. [28]
TOCNFs/CaPGO			
HA/CS	Natural	Regenerative medicine	Alessio et al. [55]
HA/BC			
FCAh	Hybrid	In vitro physiology	Dupont et al. [56]
PAh	Synthetic	In vitro pathophysiology	Serena et al. [9]
A/M	Natural	In vitro pathology	Cavo et al. [57]
PU-MC	Hybrid	Regenerative medicine	Cochis et al. [31]
F/C	Natural	In vitro physiology	Ugolini et al. [60]
PAAM	Synthetic	In vitro physiology	Orsi et al. [62]
F	Natural	In vitro physiology	Arrigoni et al. [63]
			Massai et al. [64]
C	Natural	In vitro physiology	Bono et al. [65]
Matrigel^TM^	Natural	In vitro physiology	Marasso et al. [66]

**Legend: DAC^®^:** Defensive Antibacterial Coating; **P-CHP407:** a thermo- and PH-responsive amphiphilic poly(ether urethane); **Fmoc-FF:** N^α^-fluorenylmethyloxycarbonyl diphenylalanine; **(FY)3:** hexapeptide containing three phenylalanine (F) and three tyrosine (Y) residues; **PEG_8_-(FY)3:** PEGylated version of (FY)3; **D-Leu-Phe-Phe:** tripeptide composed by D-leucine and phenylalanine; **SF:** silk fibroin; **PEGDa:** polyethylene-glycol-diacrylated; **MC:** methylcellulose; **TE:** tissue engineering; **GE:** gelatin; **MF:** methyl furfural; **CH:** chitosan; **Star-PEG-MA:** Star-PEG functionalized with maleimide groups as a dienophile; **2 PL:** two-photon lithography; **GelMA:** gelatin methacrylate; **PLU:** pluronic F-127; **GG:** gellan gum; **GO:** graphene oxide; **PF:** polyethylene glycol-fibrinogen; **TOCNFs:** TEMPO-oxidized cellulose nanofibers; **CaP:** calcium phosphate; **CaPGO:** calcium phosphate-graphene oxide; **HA:** hyaluronic acid; **CS:** chondroitin sulfate; **BC:** bio-fermentative unsulphated chondroitin; **FCAh:** fibronectin-coated acrylamide hydrogels; **Pah:** poly-acrylamide hydrogel; **A:** alginate; **M:** matrigel; **PU-MC:** polyurethane-methylcellulose; **PAAM:** polyacrylamide; **F:** fibrin; **C:** collagen type I.

## Data Availability

The data that support the reviewed articles are contained within the article and more information are available from the referenced studies.
